# The effects of hemoadsorption in patients with sepsis-associated persistent severe AKI who require CRRT: A prospective, randomized controlled pilot study

**DOI:** 10.1016/j.aicoj.2026.100167

**Published:** 2026-09-22

**Authors:** Keran Shi, Wei Jiang, Lin Song, Yuchen Wang, Xianghui Li, Jing Wang, Ying Shi, Zhixiang Yang, Xing Cai, Wei Han, Jiujiang Liu, Jingjing Yin, Xiaohua Gu, Qingquan Lyu, Xiaolan Xu, Renyan Zhao, Haixia Wang, Qiaoping Chen, Qingjie Zhu, Xiaoyan Wu, Jiangquan Yu, Ruiqiang Zheng, for the Acute Kidney Injury Precision Prevention and Treatment Research Team(AKI-PPT)

**Affiliations:** a Northern Jiangsu People's Hospital Affiliated to Yangzhou University, Yangzhou 225000, China; b Northern Jiangsu People's Hospital, Yangzhou 225000, China

**Keywords:** Persistent acute kidney injury, Sepsis, Hemoadsorption, Biomarker, Randomized controlled trial

## Abstract

**Background:**

Sepsis-associated persistent severe acute kidney injury (S-PAKI) is marked by a sustained hyperinflammatory response and is associated with a poor prognosis. Hemoadsorption functions by removing circulating cytokines and other inflammatory mediators. This study aims to evaluate the impact of CVVH combined with hemoadsorption on the prognosis of patients with S-PAKI who need CRRT.

**Methods:**

This prospective, randomized, single-center pilot study involved 76 S-PAKI patients who required CRRT. Participants were randomly assigned to receive either CVVH combined with hemoadsorption (CVVH + HA group, n = 38) or conventional CVVH therapy (CVVH group, n = 38). The primary endpoint was 30-day all-cause mortality. Secondary endpoints included changes in Acute Physiology and Chronic Health Evaluation II (APACHE II) and Sequential Organ Failure Assessment (SOFA) scores, renal functional prognosis, alterations in biomarker and cytokine levels, changes in hemodynamic parameters, as well as the duration of renal replacement therapy, mechanical ventilation, ICU stay, and overall hospital stay among survivors.

**Results:**

The CVVH + HA group showed a statistically non-significant trend to lower 30-day all-cause mortality compared to the CVVH group (36.8% vs. 52.6%, P = 0.159). A post hoc exploratory subgroup analysis revealed that among patients with Chemokine (C-C motif) ligand 14 (CCL14) levels greater than 1.3 ng/ml, the mortality risk in the CVVH + HA group was lower than that in the CVVH group. In terms of secondary outcomes, hemoadsorption therapy significantly decreased APACHE II and SOFA scores, enhanced cytokine clearance, and improved hemodynamic status; however, it did not lead to significant improvements in renal functional prognosis, nor did it shorten the duration of mechanical ventilation, renal replacement therapy, ICU stay, or overall hospital stay among survivors.

**Conclusions:**

In this pilot trial, hemoadsorption therapy did not significantly reduce 30-day mortality across the entire cohort. However, a post hoc exploratory subgroup analysis suggested a potential association with improved survival in patients with elevated urinary CCL14 levels. These exploratory, hypothesis-generating findings require confirmation in adequately powered, biomarker-guided randomized trials.

**Trial registration:**

ChiCTR2400093572, registered at the Chinese Clinical Trial Registry (ChiCTR) on December 09, 2024.

## Highlights

• This pilot study suggests for the first time that CVVH combined with hemoadsorption therapy is potentially associated with improved clinical outcomes in patients with urinary CCL14 levels exceeding 1.3 ng/ml.
• These observed associations must be strictly interpreted as hypothesis-generating and requiring confirmation in adequately powered, biomarker-guided randomized trials.
• This study also incorporated coagulation parameters, blood purification indices, and observation of other adverse events, thus verifying the safety and feasibility of the therapeutic regimen.

## Background

Sepsis-associated acute kidney injury (SA-AKI) is a common target-organ damage in sepsis [1]. Each year, approximately 50 million cases of sepsis are reported worldwide, with 50% of these patients experiencing acute kidney injury (AKI) [2,3]. The duration of AKI is associated with adverse prognostic outcomes, among which persistent severe AKI is a hallmark event for poor prognosis [4]. Persistent severe AKI is defined as stage 3 AKI diagnosed according to KDIGO criteria with a duration of ≥72 h, or requirement for renal replacement therapy (RRT), or death following the diagnosis of stage 3 AKI [5]. Although the incidence of persistent severe AKI in critically ill patients is only 5%, its mortality rate exceeds 40%, making it a major modifiable determinant of outcome [6].

With ongoing research into the pathophysiological mechanisms of SA-AKI, three primary pathogenic mechanisms have emerged: dysregulated inflammatory responses, microcirculatory dysfunction, and metabolic reprogramming [3,7]. Under normal circumstances, the host's inflammatory response to infection serves as an appropriate protective mechanism. However, in septic patients, the immune system is inappropriately activated, resulting in a dysregulated massive release of pro-inflammatory and anti-inflammatory cytokine [8]. Inflammatory mediators activate the inflammatory cascade, leading to tubular epithelial cell injury through the release of reactive oxygen species, mitochondrial damage, and oxidative stress, thereby exacerbating the rapid progression of AKI [9]. Unlike transient AKI, persistent AKI consistently displays the clinical features of a sustained inflammatory response, characterized by elevated plasma levels of inflammatory markers such as C-reactive protein (CRP), interleukin-6 (IL-6), and procalcitonin (PCT) [10,11]. Injured tubular epithelial cells and endothelial cells in a pro-inflammatory state during persistent AKI can continuously release inflammatory cytokines and damage-associated molecular patterns (DAMPs). These molecular mediators exacerbate the progression of glomerular fibrosis, leading to matrix deposition and a subsequent reduction in capillary density. This process ultimately results in maladaptive repair, manifesting as persistent renal dysfunction [12]. The sustained inflammatory response and maladaptive repair following AKI are the main reasons for its transition to acute kidney disease (AKD) and chronic kidney disease (CKD) [13]. Multiple clinical studies have found that the duration and severity of AKI are closely related to the incidence of AKD and CKD, as well as increased mortality [[14], [15], [16]]. Controlling the inflammatory cytokine storm in sepsis-associated persistent severe AKI(S-PAKI) is viewed as a promising therapeutic approach to prevent further renal damage and enhance survival rates [17,18].

Continuous renal replacement therapy (CRRT) utilizing the continuous veno-venous hemofiltration (CVVH) modality can provide renal functional support but cannot rapidly clear inflammation-related cytokines [19]. Hemoadsorption is an extracorporeal blood purification technique capable of clearing medium-sized, water-soluble toxic substances such as cytokines, pathogens, or endotoxin [20]. Several previous studies have demonstrated the significant efficacy of hemoadsorption in reducing plasma inflammatory cytokine levels [21,22]. HA380 is an adsorptive hemoperfusion cartridge composed of various highly biocompatible porous polymer beads. These beads are coated with a styrene-divinylbenzene copolymer, which ensures exceptional biocompatibility and minimal cytotoxicity[23,24]. A recent review highlights that the HA380 hemoperfusion cartridge is capable of adsorbing inflammatory cytokines and toxins with molecular weights ranging from 10 to 60 kDa onto the surface of its microbeads. This process effectively lowers the levels of plasma inflammatory mediators [25]. Combining hemoadsorption therapy with CVVH offers a dual advantage of supporting renal function while facilitating cytokine clearance. This approach theoretically has the potential to slow the progression of S-PAKI that necessitates CRRT, ultimately enhancing patient survival rates.

Currently, there is a notable absence of data concerning hemoadsorption use in patients suffering from S-PAKI who require CRRT. Consequently, this study was designed to assess the influence of hemoadsorption therapy on mortality rates among S-PAKI patients requiring CRRT. Additionally, it aimed to evaluate the therapy's effectiveness in removing inflammatory factors, stabilizing hemodynamics, and improving pertinent clinical outcomes.

## Methods

### Study design and patients

This study was a prospective, randomized, blinded, single-center clinical trial conducted at Northern Jiangsu People's Hospital, which is affiliated with Yangzhou University. The trial enrolled patients with S-PAKI requiring CRRT and were admitted to the intensive care unit (ICU). The inclusion criteria for participation were as follows: patients must be aged 18 years or older; meet the Sepsis 3.0 diagnostic criteria; be diagnosed with AKI according to the KDIGO 2012 criteria within 1–7 days after the diagnosis of sepsis; fulfill the indications for CRRT; and have plasma levels of IL-6 greater than 500 pg/ml, PCT greater than 5 ng/ml, or CRP greater than 100 mg/L. Additionally, informed consent was required from all participants. Persistent severe AKI was characterized by a confirmed diagnosis of AKI alongside a concurrent necessity for CRRT. CRRT indications included: (1) Uremia (blood urea nitrogen > 28.56 mmol/L or serum creatinine > 176.8 μmol/L); (2) Drug-resistant hyperkalemia (serum potassium level > 5.5 mmol/L); (3) Diuretic-resistant oliguria or anuria; (4) Central venous pressure (CVP) >12 mmHg, fluid overload, or pulmonary edema unresponsive to diuretics (PaO_2_/FiO_2_ < 300 mmHg); (5) Metabolic acidosis (arterial blood pH < 7.2). The baseline serum creatinine(sCr) level was established as either the median serum creatinine value recorded within the six months before admission or the lowest value noted within the seven days leading up to enrollment. If no sCr data were available before enrollment, the baseline glomerular filtration rate (GFR) was assumed to be 75 mL/min/1.73 m², and the baseline sCr level was estimated using the Modification of Diet in Renal Disease (MDRD) equation: eGFR = 186 × (sCr)^−1.154^ × (age)^−0.203^ × (0.742 for females) [26]. For patients diagnosed with stage 1-3 CKD, if there were no serum creatinine data available prior to enrollment, the initial serum creatinine measurement obtained after admission was utilized as the baseline.

The following cases were excluded: patients with allergies to resin, heparin, or protamine; individuals with pre-existing stage 4-5 CKD; patients who had received RRT prior to ICU admission; and those in terminal status. The study protocol was approved by the Ethics Committee of Northern Jiangsu People's Hospital (Approval Number: 2024ky081) and registered at the Chinese Clinical Trial Registry (ChiCTR) (Registration Number: ChiCTR2400093572).

### Randomisation and blinding

Eligible patients were randomly assigned in a 1:1 ratio to the CVVH combined with hemoadsorption group (CVVH + HA group) or the conventional CVVH therapy group (CVVH group). This study employed a permuted block randomization method with variable block sizes. The randomization sequence was generated by an independent researcher using Microsoft Excel. To ensure strict allocation concealment, sequentially numbered, opaque, and sealed envelopes were utilized. Specific group assignments were concealed within these envelopes, which were prepared and securely stored by another independent researcher. Each envelope was labeled exclusively with a unique participant sequence number. The investigator was permitted to open the envelopes sequentially only after confirming a patient's enrollment, resulting in two evenly distributed groups of 38 participants each.

Participant blinding was implemented in this trial; however, no blinding was applied to investigators, physicians, or nurses.

### Interventions

For patients in the CVVH + HA group, CRRT in CVVH mode was performed using the AN69 ST100 hemofilter (Baxter, USA) combined with hemoadsorption therapy via the HA380 hemoperfusion cartridge (Jafron, China). The protocol for the HA380 cartridge was as follows: 2 cartridges were used within 24 h after enrollment, followed by 1 cartridge per day for the next 2 days, with each cartridge used for 6 h [27]. For patients assigned to the CVVH group, CRRT in the CVVH mode was administered solely using the AN69 ST100 hemofilter (Baxter, USA). All patients received CRRT with a blood flow rate set at 150 ml/min, a replacement fluid flow rate of 1500 ml/h, a treatment dose ranging from 20 to 25 mL/kg/h, and a filtration fraction between 20% and 25%. ICU physicians followed the “Surviving Sepsis Campaign (SSC): International Guidelines for Management of Sepsis and Septic Shock 2021” and KDIGO clinical practice guidelines, and that the anticoagulation strategy, fluid removal volume, fluid management, and vasopressor dosages were dynamically adjusted based on the patients' real-time clinical conditions. If a patient's urine output exceeded 400 ml/24 h within 72 h of enrollment and acid-base and electrolyte imbalances were corrected (indicating improvement in SA-AKI), the CVVH and hemoadsorption therapy were terminated.

Adverse events (AEs) recorded in this study encompassed hemorrhage, thrombosis, infection, and complications associated with hemoadsorption and CRRT (i.e., hypotension and disturbances in the internal environment disturbance). AEs were documented throughout the enrollment period. Serious adverse events (SAEs) were characterized as those leading to death, life-threatening conditions, hospitalization or extended hospital stays, or permanent or severe dysfunction.

### Trial endpoints

The primary endpoint of this study was 30-day all-cause mortality after ICU admission. Secondary endpoints included changes in the Acute Physiology and Chronic Health Evaluation II (APACHE II) score and Sequential Organ Failure Assessment (SOFA) score at 72 h post-enrollment, renal functional prognosis, changes in biomarker levels (Chemokine (C-C motif) ligand 14 (CCL14), Neutrophil Gelatinase-Associated Lipocalin (NGAL), Kidney Injury Molecule-1 (KIM-1)), changes in cytokine levels (IFN-γ, IFN-a, TNF-a, IL-1β, IL-10, IL-8, IL-5, IL-2, IL-17, IL-4, IL-12, IL-6), changes in hemodynamic parameters (Mean Arterial Pressure (MAP), Lactic Acid (Lac), equivalent norepinephrine (NE) dose, vasoactive inotropic score (VIS)), and the duration of renal replacement therapy (RRT), mechanical ventilation, length of ICU stay, and overall length of hospital stay among survivors.

Blood and urine samples were collected from all patients at enrollment and 72 h post-enrollment, along with the recording of hemodynamic parameters and laboratory test results. The criteria for AKI recovery status within 7 days of enrollment were defined as follows: Complete recovery: The last serum creatinine test value within 7 days post-enrollment <1.2 times baseline; Partial recovery: The last serum creatinine test value within 7 days post-enrollment >1.2 times but <1.5 times baseline; No recovery: The last serum creatinine test value within 7 days post-enrollment ≥1.5 times baseline, or the patient was still receiving renal replacement therapy; Not tested: No repeat serum creatinine test was performed within 7 days post-enrollment. If a patient died within 7 days of enrollment, their AKI recovery status was classified as no recovery. If a patient died before discharge, their need for RRT at discharge was classified as 'Yes'. The criterion for renal functional recovery was a serum creatinine test value <1.5 times baseline. The criterion for persistent renal dysfunction was: Glomerular filtration rate (GFR) < 60 ml/min/1.73 m^2^ [28]. If a patient died within 30 days, they were classified as having unrecovered renal function and ongoing renal dysfunction. The status of sepsis infection, duration of CRRT use, duration of mechanical ventilation, and length of ICU stay were meticulously documented throughout the ICU admission. Additionally, the total length of hospital stay was consistently tracked for all patients during their hospitalization. Blood and urine samples from patients were promptly centrifuged at 3000 revolutions per minute (rpm) for 10 min, allowed to settle for 30 min to facilitate the separation of the supernatant, and subsequently stored at temperatures of ≤−70℃.

### Cytokine and biomarker measurement

Three urinary biomarkers (CCL14, NGAL, KIM-1) were quantified using the following enzyme-linked immunosorbent assay (ELISA) kits: Human CCL14 ELISA Kit (Boster Biological Technology, Wuhan, China. Catalog#EK1123), Human Lipocalin-2/NGAL/LCN2 ELISA Kit (Boster Biological Technology, Wuhan, China. Catalog#EK0853), Human KIM-1/HAVCR1 ELISA Kit (Boster Biological Technology, Wuhan, China. Catalog#EK0883). The quantitative detection of twelve cytokines was performed using the flow cytometric luminescence assay on the Luminex platform (Luminex Corporation, Texas, USA). All analyses were conducted in strict accordance with the manufacturer's instructions.

### Statistical analysis

As a pilot study, the primary objective of this research was to evaluate the feasibility, safety, and protocol adherence of the trial; it was not powered to detect differences in mortality. The sample size was calculated to ensure a high probability of identifying unforeseen adverse events or feasibility barriers. Using the pilot study sample size formula in the PASS software, n = ln(1 − γ)/ln(1−π), we set the confidence level (γ) at 95% and the minimum acceptable problem incidence rate (π) at 5%. This calculation indicated a minimum requirement of 59 patients. Accounting for a 10% dropout rate, at least 66 patients were required. Ultimately, the actual total sample size enrolled in this study was 76 patients, with 38 patients initially recruited per group for analysis.

Continuous data with a normal distribution were expressed as mean ± standard deviation (mean ± SD), while those with a non-normal distribution were presented as median (interquartile range) [M (P25-P75)]. Categorical data were described using frequencies and percentages. The Shapiro-Wilk test was utilized to assess the normality of data distribution. Comparisons of quantitative data between two groups were performed using the t-test for normally distributed data; otherwise, the Mann-Whitney *U* test was applied. Comparisons of categorical data between two groups were performed using the Chi-square test, Fisher's exact test, or Kruskal-Wallis test, as appropriate. Survival analysis was conducted using the Cox proportional hazards regression model to calculate the hazard ratio (HR) and 95% confidence interval (95% CI) for the treatment effect. Kaplan-Meier curves displayed the distribution of survival time, and differences in survival rates were compared using the log-rank test. A post hoc exploratory subgroup analysis was performed based on age, gender, hypertension, diabetes, APACHE II score, IL-6, CCL14, NGAL, and KIM-1. The cutoff value reported by Koyner et al. [29] was applied for CCL14. Meanwhile, the cohort's baseline medians were used as cutoffs to dichotomize age, APACHE II score, IL-6, NGAL, and KIM-1 into high and low subgroups. Each factor in the subgroup analyses was evaluated using the rate of the primary endpoint, and the interaction effects of the factors were tested using the Cox model. Interaction terms were included in the Cox model, and hypothesis testing was performed on these terms to determine whether the treatment effect varied by subgroup variables. Multivariable Cox regression analyses, adjusted for age, sex, and baseline APACHE II score, were performed, with results reported as hazard ratios (HRs) and 95% confidence intervals (CIs). Given the small sample size of this pilot study and the exploratory nature of the subgroup analyses, no multiple testing corrections were performed, which may increase the probability of Type I errors. The proportional hazards (PH) assumption for all Cox regression models was evaluated using Schoenfeld residuals.

During the peer-review process, as a methodological refinement, Linear Mixed-Effects Models (LMMs) were used to perform a post hoc re-analysis of all repeatedly measured continuous variables, including APACHE II scores, SOFA scores, biomarker levels, cytokine levels, hemodynamics, and coagulation indicators. The proportion and distribution of missing data are presented in Table S1. To avoid post hoc modifications related to competing risks of death, the duration of mechanical ventilation, RRT duration, ICU length of stay, and hospital length of stay were analyzed exclusively among 30-day survivors. A two-tailed P value < 0.05 was considered statistically significant. Statistical analyses and graphing were conducted using R 4.5.3, Stata 15, and GraphPad Prism 9.5.0 software.

## Results

### Patients

Between December 10, 2024, and April 30, 2025, a total of 126 S-PAKI patients requiring CRRT were admitted to the ICU, of whom 50 (39.7%) were not enrolled. Among those who did not meet the inclusion criteria, 2 (1.6%) were under 18 years of age, 21 (16.7%) had plasma IL-6 levels < 500 pg/ml, PCT < 5 ng/ml, and CRP < 100 mg/l, and 3 (2.4%) did not provide informed consent. The primary reasons for exclusion were pre-existing stage 4-5 CKD (n = 17, 13.5%), receipt of renal replacement therapy prior to ICU admission (n = 2, 1.6%), and being in an end-of-life state (n = 5, 4.0%). Seventy-six patients with sepsis-associated persistent severe AKI were included and randomized: 38 were allocated to the CVVH + HA group and 38 to the CVVH group (Fig. 1). There were no significant differences in baseline clinical characteristics between the two groups (Table 1).

**Fig. 1:**
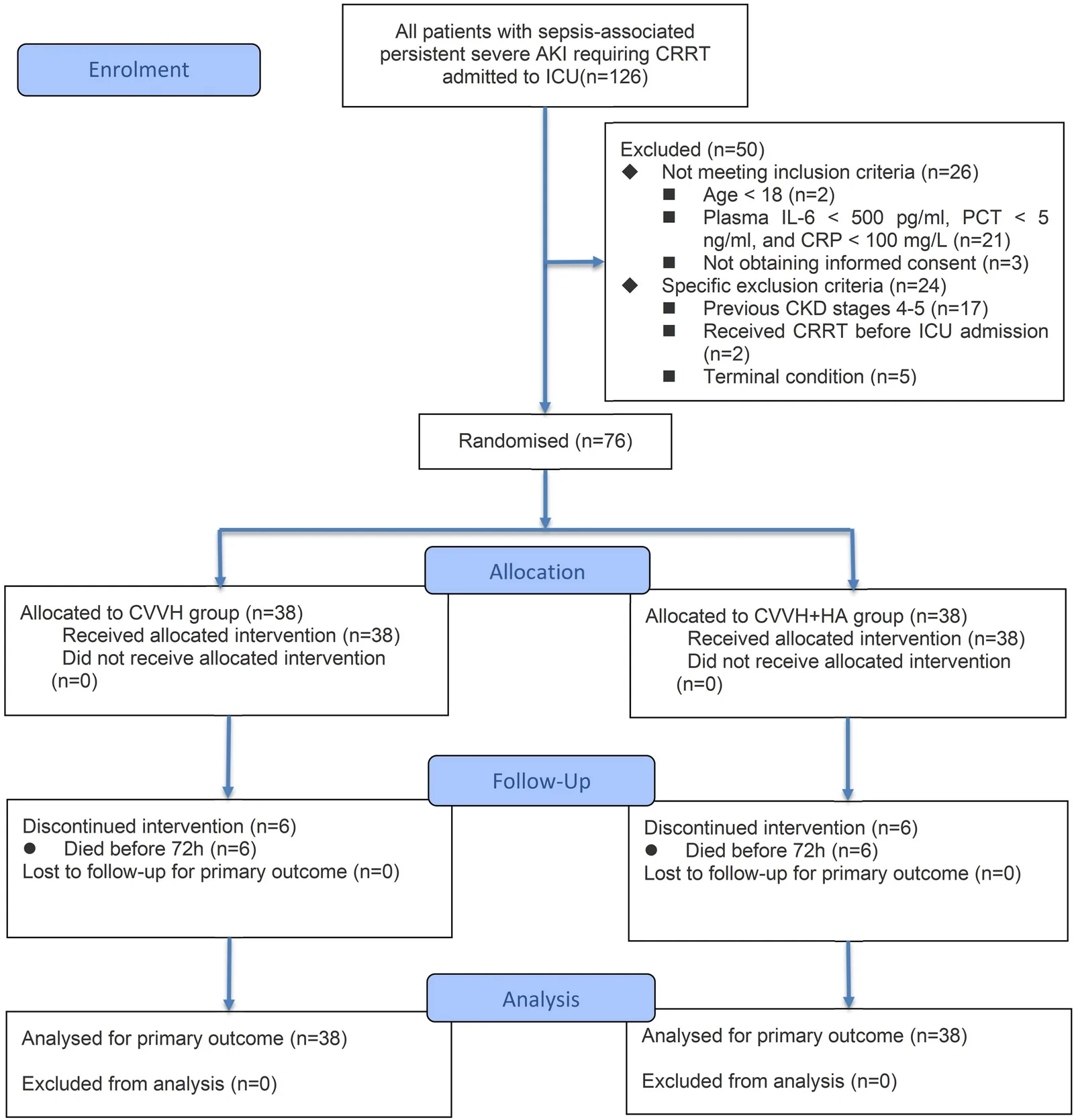
CONSORT patient flow diagram. *ICU*, intensive care unit; *CVVH*, continuous veno-venous hemofiltration; *HA*, hemoadsorption.

**Table 1:** Baseline patients’ characteristics.

	Total (n = 76)	CVVH group (n = 38)	CVVH + HA group (n = 38)	P value
Male, n (%)	51(67.1)	24(63.1)	27(71.0)	0.464
Age, years, mean ± SD	68.3.6 ± 13.7	70.3 ± 12.9	66.2 ± 14.4	0.193
BMI, kg/m^2^, mean ± SD	23.1 ± 4.2	22.6 ± 4.7	23.7 ± 3.7	0.256
Underlying Diseases, n (%)				
Diabetes	25(32.9)	10(26.3)	15(39.5)	0.222
Hypertension	39(51.3)	17(44.7)	22(57.9)	0.251
COPD	5(6.6)	2(5.3)	3(7.9)	0.644
Coronary Heart Disease	6(7.9)	3(7.9)	3(7.9)	1.000
Arrhythmia	9(11.8)	6(15.8)	3(7.9)	0.287
Cerebrovascular Disease	12(15.8)	7(18.4)	5(13.2)	0.529
CKD	6(7.9)	3(7.9)	3(7.9)	1.000
Infection Site, n (%)				
Lungs	51(67.1)	24(63.2)	27(71.1)	0.464
Abdominal Cavity	38(50.0)	19(50.0)	19(50.0)	1.000
Urinary System	19(25.0)	8(21.1)	11(29.0)	0.427
Bloodstream	38(50.0)	19(50.0)	19(50.0)	1.000
Etiological Culture Result				
Gram-positive bacteria	37(48.7)	20(52.6)	17(44.7)	0.491
Gram-negative bacteria	42(55.3)	18(47.4)	24(63.2)	0.166
Fungi	23(30.3)	10(26.3)	13(34.2)	0.454
Viruses	14(18.4)	10(26.3)	4(10.5)	0.076
Not detected	14(18.4)	6(15.8)	8(21.1)	0.554
Hemodynamic Parameters				
Lac, mmol/l, median (IQR)	3.4(2.4–5.5)	2.8(1.8–5.0)	4.6(2.8–6.2)	0.086
Equivalent Norepinephrine Dose, μg/min, median (IQR)	39.8(13.3–65.5)	30(6–59)	47.8(24–71.5)	0.058
VIS, points, median (IQR)	64.2(21.4–109.3)	51.5(10–96.3)	79.8(40–122.2)	0.060
Renal Function				
Scr, µmol/l, median (IQR)	206.2(131.6–307.4)	200(93.0–307.7)	206.2(143.0–307.1)	0.513
24-h urine output, ml, median (IQR)	464.0(205.5–828.5)	439.5(216.0–1044.0)	473.0(176.0–779.0)	0.848
Cytokines				
IL-6, pg/ml, median (IQR)	1016.4(213.1–2594.0)	471.0(106.7–2071.1)	1115.9(501.4–2833.5)	0.064
IL-8, pg/ml, median (IQR)	113.4(37.7–350.2)	111.8(23.9–440.2)	118.5(57.6–311.9)	0.716
IL-10, pg/ml, median (IQR)	13.7(3.7–84.0)	8.6(3.5–39.1)	30.1(4.8–110.5)	0.079
IFN-γ, pg/ml, median (IQR)	10.7(5.7–27.2)	9.3(5.3–18.7)	11.8(6.0–36.4)	0.400
IFN-α, pg/ml, median (IQR)	1.8(1.4–2.2)	1.6(1.4–2.1)	1.8(1.4–2.3)	0.397
IL-1β, pg/ml, median (IQR)	10.5(6.4–20.4)	10.9(6.1–19.6)	9.3(6.5–21.2)	0.950
TNF-α, pg/ml, median (IQR)	1.6(1.6–2.2)	1.6(1.6–2.1)	1.7(1.6–2.6)	0.168
Biomarkers				
CCL14, ng/ml, median (IQR)	1.5(0.5-2.6)	1.6(0.5-2.5)	1.5(0.6-2.8)	0.513
NGAL, ng/ml, median (IQR)	177.0(78.4-307.0)	159.3(75.7-307.9)	212.5(85.9-306.2)	0.547
KIM-1, ng/ml, median (IQR)	3.1(1.2-8.5)	4.3(0.9-8.7)	2.3(1.3-8.2)	0.533

CVVH, continuous veno-venous hemofiltration; HA, hemoadsorption; BMI, body mass index; COPD, chronic obstructive pulmonary disease; CKD, chronic kidney disease; Lac, lactic acid; VIS, vasoactive inotropic score; Scr, Serum Creatinine; PCT, procalcitonin; IL, interleukin; IFN, interferon; TNF, tumor necrosis factor; CCL14, chemokine (c-c motif) ligand 14; NGAL, neutrophil gelatinase-associated Llipocalin; KIM-1, kidney injury molecule-1.

### Feasibility and safety

Among patients randomized to the CVVH + HA group, hemoadsorption was initiated with a median delay of 19 (6, 27) h after ICU admission and 2 (1, 18) h after enrollment. According to the treatment protocol, 17 patients received 4 sessions of HA within 72 h, 5 patients received 3 sessions, 10 patients received 2 sessions, and 6 patients received 1 session within 72 h. Of these, 15 patients did not complete 4 sessions of HA due to improved renal function, while 6 patients were unable to complete 4 sessions due to death.

Regarding coagulation safety assessment, no significant differences were observed in prothrombin time (PT), thrombin time (TT), activated partial thromboplastin time (APTT), international normalized ratio (INR), or fibrinogen (FIB) levels between enrollment and 72 h post-enrollment in the CVVH + HA group (Table S2). Additionally, after enrollment, no other adverse events related to hemoadsorption, such as bleeding, thrombosis, infection, or disturbances in the internal milieu, were observed.

### CRRT and hemoadsorption management and ICU care

There were no significant differences between the two groups regarding anticoagulation strategies and medications for CRRT and hemoadsorption. When the CVVH mode was configured for CRRT in all patients, the blood flow rate was set to 150 ml/min, and the replacement fluid rate was set to 1500 ml/h. Other CRRT parameters, such as hourly ultrafiltration volume, were comparable between the two groups. Furthermore, respiratory support modalities, shock status, SOFA scores, and APACHE II scores at baseline also showed no significant intergroup differences (Table 2).

**Table 2:** Intensive care unit, CRRT and hemoadsorption management.

	Total (n = 76)	CVVH group (n = 38)	CVVH + HA group (n = 38)	P value
Anticoagulant drugs, n (%)				
Regional citrate anticoagulant	69(90.8)	35(92.1)	34(89.5)	1.000
Unfractionated heparin	8(10.5)	4(10.5)	4(10.5)	1.000
Argatroban	2(2.6)	2(5.26)	0(0)	0.493
No anticoagulation	10(13.2)	6(15.8)	4(10.5)	0.497
CRRT fluid removal volume, ml/h, median (IQR)				
Day 1 Max Fluid Removal	244.0(178.0–299.5)	252.5(178.0–299.0)	236.5(178.0–300.0)	0.670
Day 2 Max Fluid Removal	201.0(121.0–296.0)	194.0(144.0–293.0)	201.5(117.0–296.0)	0.725
Day 3 Max Fluid Removal	193.0(124.0–248.0)	194.5(130.0–279.0)	192.5(122.0–246.0)	0.973
Respiratory Support, n (%)				0.417
Nasal cannula oxygen	7(9.2)	3(7.9)	4(10.5)	
High-flow nasal oxygen	8(10.5)	2(5.3)	6(15.8)	
Non-invasive ventilation	7(9.2)	3(7.9)	4(10.5)	
Invasive mechanical ventilation	54(71.1)	30(79.0)	24(63.2)	
Shock, n (%)	66(86.8)	32(84.2)	34(89.5)	0.497
SOFA Score, mean ± SD	13.2 ± 3.2	12.8 ± 3.4	13.6 ± 3.1	0.292
APACHE II Score, mean ± SD	30.4 ± 6.5	29.0 ± 7.7	31.8 ± 4.8	0.065

CVVH, continuous veno-venous hemofiltration; HA, hemoadsorption; CRRT, continuous renal replacement therapy; SOFA, Sequential Organ Failure Assessment; APASCHE II, Acute Physiology And Chronic Health Evaluation II.

### Primary endpoints

The primary endpoint of this study was 30-day all-cause mortality following ICU admission. During the follow-up period, 14 patients (36.8%) in the CVVH + HA group and 20 patients (52.6%) in the CVVH group died. Kaplan-Meier survival curve analysis revealed that while combined hemoadsorption therapy resulted in an absolute mortality reduction of 15.8%, there was no statistically significant difference in overall survival between the two groups (Fig. 2).

**Fig. 2:**
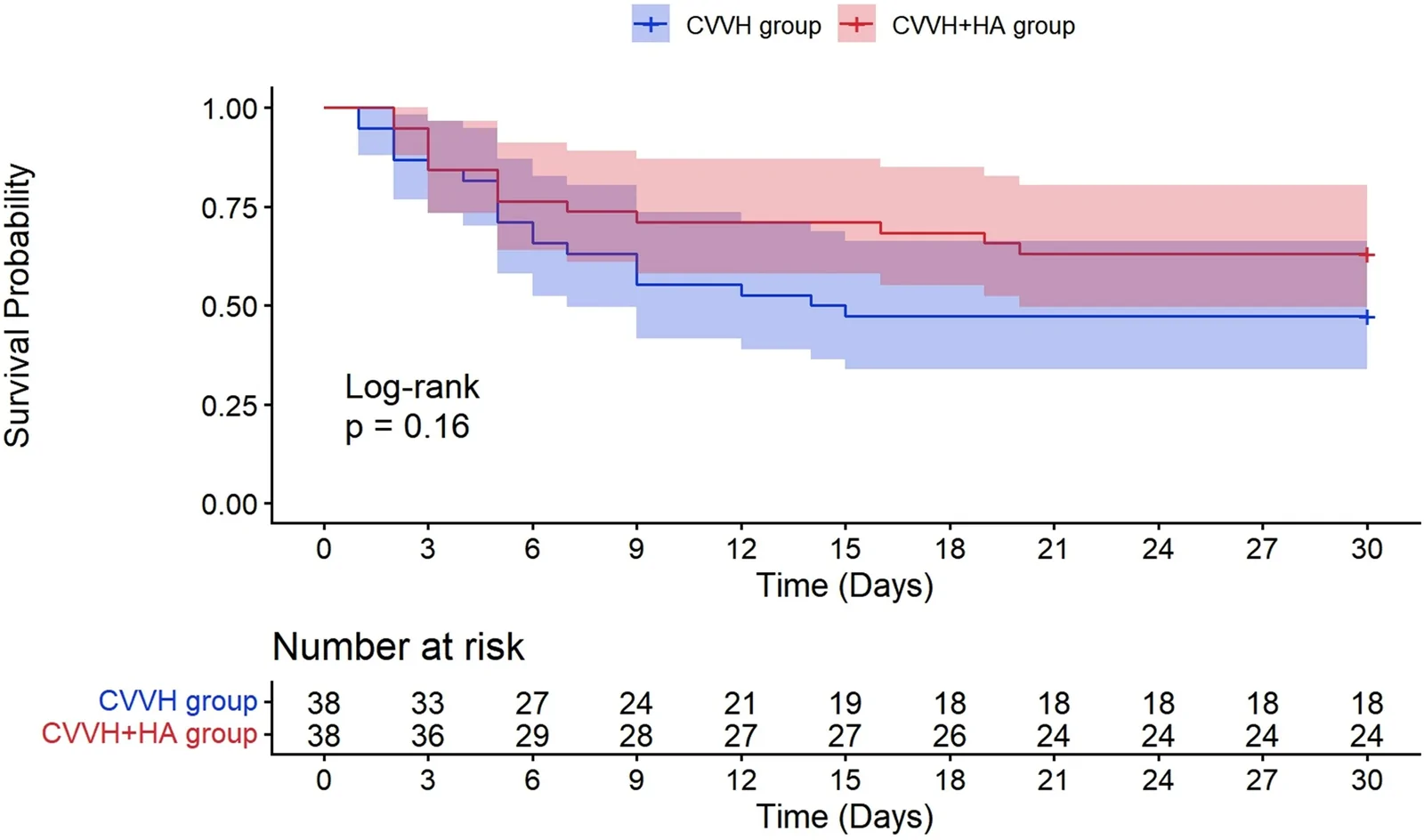
The Kaplan–Meier estimates of mortality at 30 days. *CVVH*, continuous veno-venous hemofiltration; *HA*, hemoadsorption.

### Subgroup analysis

Post hoc analyses were conducted across 9 subgroups, including age, gender, underlying diseases, APACHE II score, IL-6, and biomarkers. The results indicated that the treatment effect remained directionally consistent across the majority of these subgroups. Notably, the intervention effect of hemoadsorption therapy showed a significant statistical interaction within the CCL14 subgroup (P for interaction = 0.011). Among patients with baseline urinary CCL14 levels greater than 1.3 ng/ml, the 30-day mortality risk in the CVVH + HA group was significantly lower than in the CVVH group (HR = 0.31, 95% CI: 0.12–0.76). In other subgroup stratifications, hemoadsorption did not have a statistically significant impact on mortality risk (Table 3). Results of the multivariable Cox regression analysis, adjusted for age, sex, and APACHE II score, demonstrated that the survival benefit persisted in the subgroup with urinary CCL14 > 1.3 ng/ml. Interestingly, after adjusting for these potential confounders, the outcomes in the subgroups with IL-6 > 1016.4 pg/ml, NGAL < 177 ng/ml, and KIM-1 < 3.1 ng/ml also suggested a potential survival benefit; however, no statistically significant interaction was observed (Table S3). The Schoenfeld residual test results indicated that both the overall cohort and all interaction models met the proportional hazards assumption requirement (Table S4).

**Table 3:** Subgroup analysis of the effect of CVVH + HA versus CVVH on 30-day mortality.

The forest plot shows unadjusted hazard ratios (HRs) and 95% confidence intervals (CIs). Blue squares and the red diamond indicate point estimates for subgroups and the overall cohort, respectively. The vertical dashed line indicates an HR of 1.0. P values for interaction indicate heterogeneity of the treatment effect across strata. All analyses were performed without adjustment for other covariates. CVVH, continuous veno-venous hemofiltration; HA, hemoadsorption; CI, confidence interval; APACHE II, Acute Physiology And Chronic Health Evaluation II; IL, interleukin; CCL14, chemokine (c–c motif) ligand 14; NGAL, neutrophil gelatinase-associated lipocalin; KIM-1, kidney injury molecule-1.

### Secondary endpoints

In analyzing secondary endpoints, linear mixed-effects models revealed that patients in the CVVH + HA group experienced a more pronounced decline in APACHE II and SOFA scores within the first 72 h of enrollment, achieving lower absolute scores at the 72-h mark (Fig. 3A, Table S5). When assessing renal functional outcomes, the recovery of renal function within 7 days post-enrollment and the rate of RRT dependence at discharge were found to be comparable between the two groups. Subsequent 30-day follow-up results indicated similar renal functional recovery for both groups (Table 4). Examining urinary biomarkers, no significant differences were observed in the absolute levels at the 72-h point or in the rates of decline over 72 h for CCL14 and KIM-1 between the groups. Notably, the CVVH + HA group showed a favorable trend in the magnitude of decline for CCL14. Conversely, NGAL and KIM-1 exhibited a more rapid downward trend in the CVVH group, which also presented with lower NGAL levels at 72 h post-enrollment (Fig. 3B and Table S5). Regarding cytokines, the CVVH + HA group exhibited lower absolute levels of IL-1β at the 72-h time point compared with the CVVH group. Relative to CVVH therapy, the combined hemoadsorption treatment demonstrated a greater magnitude of cytokine clearance, which was primarily reflected in the levels of IFN-α, TNF-α, IL-10, IL-17, and IL-6 (Fig. 3C and Table S5). In terms of hemodynamic parameters, hemoadsorption therapy facilitated a more rapid reduction in Lac levels, VIS, and equivalent NE doses, alongside an increase in MAP (Fig. 3D and Table S5). Finally, there were no statistically significant differences between the two groups regarding clinical outcome indicators, including the duration of mechanical ventilation, duration of renal replacement therapy, length of ICU stay, and overall hospital stay among survivors (Fig. 4).

**Fig. 3:**
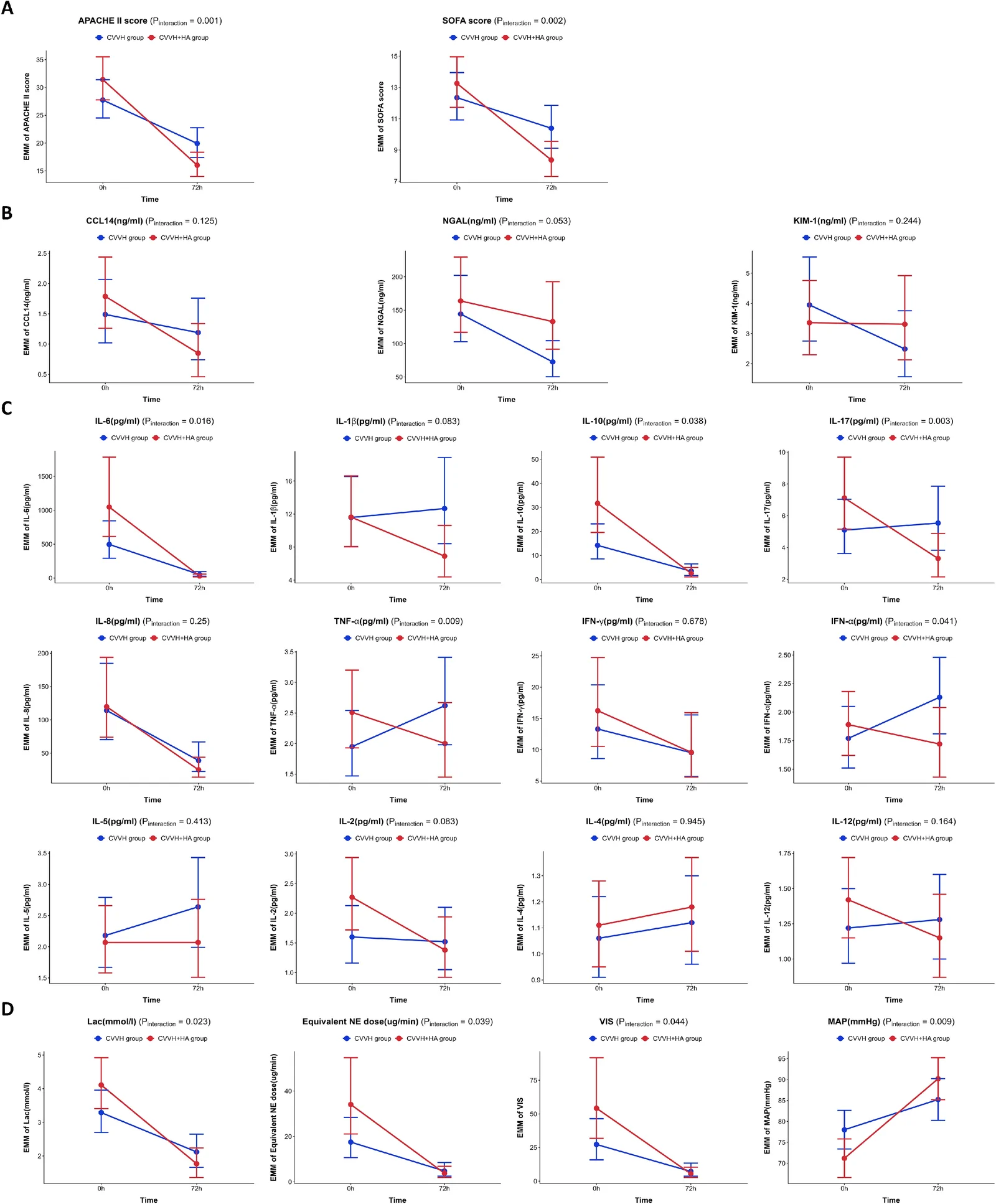
Longitudinal trajectories of secondary endpoints throughout the 72-h study period are illustrated as follows: (A) Severity scores; (B) Biomarkers; (C) Cytokines; (D) Hemodynamic parameters. The data is presented as EMMs, with error bars representing 95% CIs. All estimates and statistical analyses were derived from LMMs. LMM, linear mixed-effects model; EMM, estimated marginal mean; CI, confidence interval; CVVH, continuous veno-venous hemofiltration; HA, hemoadsorption; APACHE II, Acute Physiology And Chronic Health Evaluation II; SOFA, Sequential Organ Failure Assessment; CCL14, chemokine (c-c motif) ligand 14; NGAL, neutrophil gelatinase-associated lipocalin; KIM-1, kidney injury molecule-1; IFN, interferon; TNF, tumor necrosis factor; IL, interleukin; MAP, mean arterial pressure; Lac, lactate; VIS, vasoactive inotropic score; NE, norepinephrine.

**Table 4:** Prognosis of renal function.

	Total (n = 76)	CVVH group (n = 38)	CVVH + HA group (n = 38)	P value
Recovery status within 7 days after enrollment, n (%)				0.604
Complete recovery	26(34.2)	14(36.9)	12(31.6)	
Partial recovery	4(5.3)	1(2.6)	3(7.9)	
Unrecovered	46(60.5)	23(60.5)	23(60.5)	
Not tested	0(0)	0(0)	0(0)	
Requirement for RRT at discharge, n (%)	39(51.3)	22(57.9)	17(44.7)	0.251
Renal function recovery on Day 30, n (%)	24(31.6)	11(29.0)	13(34.2)	0.622
Persistent renal insufficiency on Day 30, n (%)	46(60.5)	24(63.2)	22(57.9)	0.639

CVVH, continuous veno-venous hemofiltration; HA, hemoadsorption; RRT, renal replacement therapy.

**Fig. 4:**
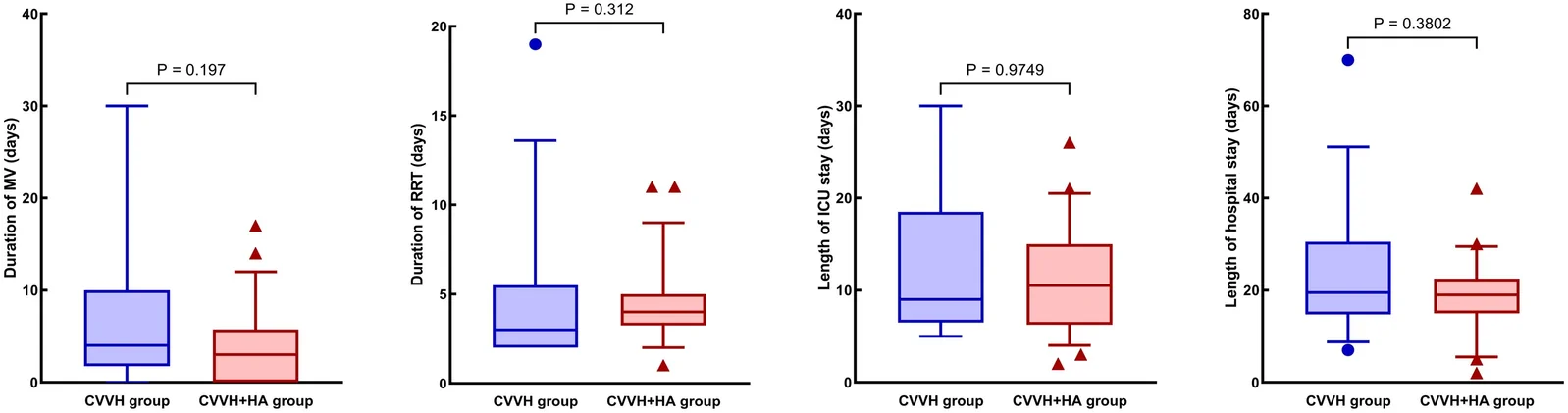
Duration of mechanical ventilation (MV), duration of RRT, length of ICU stay, and length of hospital stay are presented for survivors. Box plots illustrate the distribution of clinical time-to-event outcomes for these patients. The horizontal line within each box represents the median, while the upper and lower borders indicate the interquartile range (IQR; 25th to 75th percentiles). The upper and lower whiskers extend to the 90th and 10th percentiles, respectively, with individual points beyond the whiskers representing outliers. P values indicate the statistical significance of the differences between the two groups, calculated using the Mann-Whitney U test. CVVH, continuous veno-venous hemofiltration; HA, hemoadsorption; MV, mechanical ventilation; RRT, renal replacement therapy; ICU, intensive care unit.

## Discussion

This study demonstrated that in patients with S-PAKI requiring CRRT, the use of combined hemoadsorption therapy significantly enhanced the clearance of cytokines, leading to improved early hemodynamic status and reduced disease severity and organ dysfunction. However, we did not find a significant effect of hemoadsorption on survival outcomes or other clinical indicators, including renal functional prognosis. Notably, a post hoc exploratory subgroup analysis indicated that the potential survival benefit of hemoadsorption therapy may be associated with elevated CCL14 levels.

While we observed early physiological improvements following combined hemoadsorption, these improvements did not correspond to significant changes in patient-centered clinical outcomes, especially survival rates. This gap between biological effects and clinical outcomes raises questions about the clinical significance of the observed reductions in inflammatory biomarkers. The theoretical foundation for advocating early cytokine clearance is based on the peak concentration hypothesis. However, a more nuanced understanding of this hypothesis suggests that the peak release of pro-inflammatory cytokines, rather than simply elevated cytokine levels, is critical for causing host tissue damage and organ dysfunction [30]. Initiating intervention during the appropriate inflammatory state of the body may be the key to achieving the clinical benefits of cytokine clearance therapy. Secondly, in cases of sepsis, elevated tissue interstitial concentrations of cytokines play a critical role in causing organ damage, whereas extracorporeal cytokine clearance primarily reduces circulating cytokine levels [31]. Thirdly, current non-selective hemoadsorption devices clear not only cytokines but also components such as coagulation factors, antibiotics, and immunoglobulins, potentially exacerbating immune dysfunction and disease severity [32]. In the present study, hemoadsorption therapy did not yield significant clinical benefits in terms of renal recovery, duration of RRT, and length of hospital stay. A potential explanation for this observation could be the delayed initiation of treatment. By the time patients with S-PAKI are admitted to the ICU, substantial parenchymal damage to the kidneys has largely already occurred. Because tissue regeneration and repair require their own physiological time courses, hemoadsorption, while assisting in the clearance of inflammatory mediators, cannot rapidly restore renal function. Furthermore, the clinical outcomes of critically ill patients are influenced by the complex interplay of multiple factors, including secondary infections, nutritional status, and baseline characteristics. The addition of hemoadsorption therapy alone targets only a single pathophysiological pathway. Compared with the control group, patients receiving hemoadsorption therapy tended to present with worse baseline characteristics. This imbalance may have also acted as a confounding factor, thereby offsetting the potential clinical benefits of hemoadsorption. Nevertheless, the clinical significance of the observed reductions in inflammatory biomarkers remains important. The physiological stabilization resulting from decreased inflammatory biomarkers can provide a crucial window of opportunity and support for source control in patients with sepsis. Previous studies have suggested that the application of hemoadsorption therapy in patients with abdominal septic shock following emergency surgery for source control may improve survival outcomes and reverse organ failure [33].

In several randomized controlled trials evaluating hemoadsorption therapy in sepsis, most failed to show that hemoadsorption confers a survival benefit. One notable multicenter RCT included 243 patients suffering from septic shock due to peritonitis. In this trial, early treatment with polymyxin B hemoadsorption did not lead to a reduction in 28-day mortality or an improvement in organ dysfunction when compared to conventional therapy [34]. Similarly, the EUPHRATES trial showed that polymyxin B hemoadsorption did not demonstrate a survival advantage in patients with septic shock and endotoxemia [35]. Subsequent trials also produced unfavorable clinical results. A randomized controlled trial conducted by Schädler et al. evaluated 97 mechanically ventilated patients with sepsis. While the study confirmed a transient reduction in IL-6 levels, no significant differences in plasma IL-6 concentrations were observed between the groups. Furthermore, patients undergoing hemoadsorption exhibited significantly higher unadjusted 60-day mortality rates and a greater proportion requiring renal replacement therapy [36]. The results of a recent randomized controlled trial involving 31 patients with septic shock and a hyperinflammatory state suggested that early use of hemoadsorption therapy neither modulated the immune response nor improved clinical outcomes [37]. The negative outcomes of these RCTs indicate that the routine use of hemoadsorption in sepsis is not justified. However, a post hoc exploratory analysis of the EUPHRATES trial found that septic shock patients with baseline endotoxin activity of 0.6-0.89 units experienced significant benefits and a favorable safety profile following hemoadsorption intervention [38]. Building upon the post-hoc analysis of the EUPHRATES trial, the Tigris trial, guided by endotoxin activity, explored for the first time the efficacy of precision hemoadsorption in patients with a specific septic shock endotype. The study suggested a high probability that polymyxin B hemoadsorption is associated with a survival benefit in septic shock patients with high endotoxin activity, particularly regarding a lower 90-day mortality [39]. The findings from the Tigris trial indicate that hemoadsorption therapy is unlikely to confer a universal benefit across all patients; rather, utilizing reliable biomarkers for patient enrichment may be a prerequisite for achieving clinical benefits with this therapy. Our findings similarly suggest that the indiscriminate application of hemoadsorption in unselected patients is insufficient to reverse the clinical trajectory. Only through precise hemoadsorption therapy tailored to specific patient phenotypes can the reduction in inflammatory markers potentially translate into meaningful clinical outcomes. Therefore, the approach to hemoadsorption therapy should shift from a generalized "treat-all-sepsis" strategy to a more precision-guided treatment model.

SA-AKI, a frequent indicator of organ dysfunction in sepsis, represents a highly heterogeneous clinical syndrome [40,41]. In response to this high heterogeneity, utilizing novel biomarkers to guide SA-AKI treatment has emerged as a new strategy for precision medicine [42]. Studies suggest employing biomarkers and prognostic enrichment strategies to identify patient subgroups at high risk for adverse clinical outcomes, such as mortality and AKI. This approach aims to predict which individuals may benefit from hemoadsorption therapy, thereby enabling the selection of suitable patients for treatment [[43], [44], [45], [46]]. CCL14 is a novel inflammatory biomarker from the CC chemokine family that has shown tremendous application value in the clinical diagnosis and treatment of SA-AKI [47]. Research conducted by Koyner et al. established that a cutoff value of 1.3 ng/ml for urinary CCL14 demonstrates high sensitivity in identifying patients with persistent severe AKI. Furthermore, urinary CCL14 levels exceeding 1.3 ng/ml are significantly associated with an elevated risk of mortality [29]. A European consensus meeting recommended making timely and targeted clinical decisions in AKI management based on CCL14 levels [48]. In SA-AKI, CCL14 is predominantly secreted in large amounts by tubular epithelial cells in response to inflammatory stimulation. This release occurs mainly through the luminal side into the urine, leading to significantly increased concentrations of CCL14 in the urine [49,50]. Based on these clinical application outcomes and theoretical mechanisms, CCL14 is a reliable candidate biomarker for SA-AKI. The results of the post hoc exploratory subgroup analysis in this study are encouraging. The results of the subgroup analysis suggest that the potential survival benefit of hemoadsorption may be associated with elevated CCL14 levels. We need to clarify that the observed association in the subgroup should strictly be regarded as hypothesis-generating and requiring confirmation in adequately powered, biomarker-guided randomized trials. Hemoadsorption shows a faster trend in clearing CCL14, which could theoretically interrupt the inflammatory cascade within tissues and reduce tubular injury. However, the trajectories of the renal injury biomarkers were contrary to our expectations, as we observed a slower decline and higher absolute values at 72 h in the hemoadsorption group. We hypothesize that this may be attributed to the greater baseline disease severity and higher initial biomarker levels among patients receiving hemoadsorption. This implies a more profound degree of intrinsic kidney injury in this cohort, potentially leading to a more protracted renal recovery process in the short term.

This study presents a novel research hypothesis for the precision treatment of S-PAKI guided by the biomarker CCL14 and provides a crucial reference for designing future prospective trials. Furthermore, this study incorporated coagulation indices, blood purification parameters, and observations of other adverse events, confirming the safety and feasibility of the treatment protocol. When interpreting our results, it is important to note that the definition of persistent AKI remains unstandardized. While recent criteria define persistent AKI as a diagnosis of acute kidney injury lasting more than 48 h, other clinical studies use a threshold of 72 h or more [51]. Our study defined persistent severe AKI as a diagnosis of AKI with a concurrent need for CRRT. This definitional variation should be considered when comparing our outcomes with other hemoadsorption trials.

However, our study has several limitations. First, this study is a pilot trial with a small sample size and a trend toward baseline imbalances. Despite randomization, a trend toward worse baseline characteristics was observed in the intervention group, manifesting as higher IL-6 and IL-10 levels, higher APACHE II scores, as well as a greater requirement for vasoactive drugs. This unbalanced baseline trend may have masked the actual benefits of the intervention. Consequently, the study was underpowered to evaluate survival outcomes; nevertheless, it can serve as a reference for sample size estimation in future studies. Second, we implemented a fixed hemoadsorption treatment protocol for each patient, which may not achieve optimal therapeutic effects. The ideal timing, dose, and duration of hemoadsorption for sepsis-associated persistent severe AKI remain unknown. Third, during the treatment period, we did not perform dynamic monitoring of indicators, which precluded us from acquiring the effect trajectory of hemoadsorption on cytokine clearance. Our results showed similar levels of all cytokines at the 72-h time point between the two groups. This may be due to the natural evolution of cytokine kinetics, as levels may have already reached a physiological plateau by 72 h. The critical time point for revealing intergroup differences might be 24 or 48 h post-hemoadsorption. Results from linear mixed-effects models confirmed that, after adjusting for baseline levels, hemoadsorption still provided significantly accelerated clearance compared to the natural clearance process, which could rapidly shorten the duration patients are exposed to high inflammatory peaks. Additionally, while there was a statistically significant intergroup difference in the magnitude of TNF-α decline, this minute reduction is not clinically relevant. In such cases, the capacity of hemoadsorption to confer clinical benefits via cytokine clearance is limited. Fourth, the single-blind design of this study may lead to performance bias, especially regarding outcomes that rely on the subjective judgment of clinicians. This includes assessments of hemodynamic parameters, doses of vasoactive drugs, duration of RRT, and length of hospital stays. Fifth, the subgroup analyses conducted in this study were not pre-specified. The relationship between biomarker levels and therapeutic efficacy was identified solely through post hoc exploratory analyses, meaning these findings are intended strictly for hypothesis generation.

## Conclusion

The results of this pilot study suggest that hemoadsorption did not significantly decrease 30-day mortality rates in the overall cohort. Given the limited sample size, the study lacked sufficient power to identify any mortality differences. A post hoc exploratory subgroup analysis suggested a potential association between hemoadsorption therapy and improved clinical outcomes among patients with urinary CCL14 levels exceeding 1.3 ng/ml. However, these observed associations must be strictly presented as hypothesis-generating and requiring confirmation in adequately powered, biomarker-guided randomized trials.

## Author contributions

Ruiqiang Zheng, Jiangquan Yu contributed to the research guidance, coordination. Wei Jiang, Keran Shi and Lin Song contributed study design. Ying Shi, Zhixiang Yang, Xing Cai, Wei Han, Jiujiang Liu, Jingjing Yin, Xiaohua Gu and Qingquan Lyu participated in patient enrollment and obtaining informed consent forms. Xiaolan Xu, Renyan Zhao, Haixia Wang, Qiaoping Chen, Qingjie Zhu and Xiaoyan Wu were responsible for project supervision. Xianghui Li, Yuchen Wang and Jing Wang was responsible for data collation and data analysis. Keran Shi and Wei Jiang authored the manuscript. All authors reviewed and edited the manuscript.

## Ethics approval and consent to participate

This study adheres to the Helsinki Declaration. The study protocol was approved by the Medical Ethics Committee of Northern Jaingsu People's Hospital in April 2024 (approval number: 2024ky081) and registered with the Chinese Clinical Trial Registry (CHICTR) in December 2024 (registration number: ChiCTR2400093572). All participants or their legally authorized representatives signed written informed consent forms.

## Consent for publication

Not applicable.

## Funding

This study was supported by Jiangsu Province Frontier Technology Research and Development Plan (BF2025639); Clinical Trials Fund of Northern Jiangsu People's Hospital (Grant No. SBLC25001); The 8th Batch of Support Projects of Northern Jiangsu People's Hospital (FCJS202547); Innovative Research Project on Hospital Management (JSYGY-1-2025-660); National key clinical specialty, Financial Appropriations of National (No. 176[2022]); Flagship institution of Chinese and Western medicine coordination, Financial Appropriations of National (No. Jiangsu 60[2023]); Jiangsu Provincial Medical Key Discipline Cultivation Unit (JSDW20221); Yangzhou Municipal Science and Technology Bureau (No. YZ2024098).

## Data availability

Data from this study are available by contacting the corresponding author.

## Declaration of competing interest

None.

## References

[1] Poston J.T., Koyner J.L. (2019). Sepsis associated acute kidney injury. Bmj.

[2] Rudd K.E., Johnson S.C., Agesa K.M., Shackelford K.A., Tsoi D., Kievlan D.R. (2020). Global, regional, and national sepsis incidence and mortality, 1990-2017: analysis for the Global Burden of Disease Study. Lancet.

[3] Peerapornratana S., Manrique-Caballero C.L., Gómez H., Kellum J.A. (2019). Acute kidney injury from sepsis: current concepts, epidemiology, pathophysiology, prevention and treatment. Kidney Int.

[4] Kellum J.A., Sileanu F.E., Bihorac A., Hoste E.A., Chawla L.S. (2017). Recovery after acute kidney injury. Am J Respir Crit Care Med.

[5] Hoste E., Bihorac A., Al-Khafaji A., Ortega L.M., Ostermann M., Haase M. (2020). Identification and validation of biomarkers of persistent acute kidney injury: the RUBY study. Intensive Care Med.

[6] Gómez H., Chen X., Minturn J.S., Saraiva I.E., Hamahata N., Zaidi A. (2025). Persistent severe acute kidney injury is a major modifiable determinant of outcome during critical illness. Intensive Care Med.

[7] Gomez H., Ince C., De Backer D., Pickkers P., Payen D., Hotchkiss J., Kellum J.A. (2014). A unified theory of sepsis-induced acute kidney injury: inflammation, microcirculatory dysfunction, bioenergetics, and the tubular cell adaptation to injury. Shock.

[8] Zarbock A., Nadim M.K., Pickkers P., Gomez H., Bell S., Joannidis M. (2023). Sepsis-associated acute kidney injury: consensus report of the 28th Acute Disease Quality Initiative workgroup. Nat Rev Nephrol.

[9] Kuwabara S., Goggins E., Okusa M.D. (2022). The pathophysiology of sepsis-associated AKI. Clin J Am Soc Nephrol.

[10] Bidar F., Peillon L., Bodinier M., Venet F., Monneret G., Lukaszewicz A.C. (2024). Immune profiling of critically ill patients with acute kidney injury during the first week after various types of injuries: the REALAKI study. Crit Care.

[11] Uhel F., Peters-Sengers H., Falahi F., Scicluna B.P., van Vught L.A., Bonten M.J. (2020). Mortality and host response aberrations associated with transient and persistent acute kidney injury in critically ill patients with sepsis: a prospective cohort study. Intensive Care Med.

[12] Samoni S., De Rosa S., Ronco C., Castellano G. (2023). Update on persistent acute kidney injury in critical illnesses. Clin Kidney J.

[13] Zhang T., Widdop R.E., Ricardo S.D. (2024). Transition from acute kidney injury to chronic kidney disease: mechanisms, models, and biomarkers. Am J Physiol Renal Physiol.

[14] Meersch M., Weiss R., Strauß C., Albert F., Booke H., Forni L. (2024). Acute kidney disease beyond day 7 after major surgery: a secondary analysis of the EPIS-AKI trial. Intensive Care Med.

[15] Jensen S.K., Heide-Jørgensen U., Gammelager H., Birn H., Christiansen C.F. (2024). Acute kidney injury duration and 20-year risks of CKD and cardiovascular disease. Kidney Int Rep.

[16] Veltkamp D.M.J., Porras C.P., Gant C.M., Groenestege W.M.T., Kok M.B., Verhaar M.C. (2025). Long-term risks of adverse kidney outcomes after acute kidney injury: a systematic review and meta-analysis. Nephrol Dial Transplant.

[17] Fajgenbaum D.C., June C.H. (2020). Cytokine storm. N Engl J Med.

[18] Waalders N.J.B., Kox M., Pickkers P. (2025). Haemoadsorption to remove inflammatory mediators in sepsis: past, present, and future. Intensive Care Med Exp.

[19] Cole L., Bellomo R., Hart G., Journois D., Davenport P., Tipping P., Ronco C. (2002). A phase II randomized, controlled trial of continuous hemofiltration in sepsis. Crit Care Med.

[20] Bellomo R., Ankawi G., Bagshaw S.M., Baldwin I., Basu R., Bottari G. (2024). Hemoadsorption: consensus report of the 30th Acute Disease Quality Initiative workgroup. Nephrol Dial Transplant.

[21] Broman M.E., Hansson F., Vincent J.L., Bodelsson M. (2019). Endotoxin and cytokine reducing properties of the oXiris membrane in patients with septic shock: a randomized crossover double-blind study. PLoS One.

[22] Villa G., Romagnoli S., De Rosa S., Greco M., Resta M., Pomarè Montin D. (2020). Blood purification therapy with a hemodiafilter featuring enhanced adsorptive properties for cytokine removal in patients presenting COVID-19: a pilot study. Crit Care.

[23] Pomarè Montin D., Ankawi G., Lorenzin A., Neri M., Caprara C., Ronco C. (2018). Biocompatibility and cytotoxic evaluation of new sorbent cartridges for blood hemoperfusion. Blood Purif.

[24] Nierhaus A., Morales J., Wendt D., Scheier J., Gutzler D., Jarczak D. (2022). Comparison of the CytoSorb(®) 300 mL and Jafron HA380 hemoadsorption devices: an in vitro study. Minim Invasive Ther Allied Technol.

[25] Li Y., Han M., Yang M., Su B. (2025). Hemoperfusion with the HA330/HA380 Cartridge in Intensive Care Settings: a State-Of-The-Art review. Blood Purif.

[26] Qian B.S., Jia H.M., Weng Y.B., Li X.C., Chen C.D., Guo F.X. (2023). Analysis of urinary C-C motif chemokine ligand 14 (CCL14) and first-generation urinary biomarkers for predicting renal recovery from acute kidney injury: a prospective exploratory study. J Intensive Care.

[27] Ronco C., Reis T., De Rosa S. (2020). Coronavirus Epidemic and Extracorporeal Therapies in Intensive Care: si vis pacem para bellum. Blood Purif.

[28] Chawla L.S., Bellomo R., Bihorac A., Goldstein S.L., Siew E.D., Bagshaw S.M. (2017). Acute kidney disease and renal recovery: consensus report of the Acute Disease Quality Initiative (ADQI) 16 Workgroup. Nat Rev Nephrol.

[29] Koyner J.L., Chawla L.S., Bihorac A., Gunnerson K.J., Schroeder R., Demirjian S. (2022). Performance of a Standardized Clinical Assay for Urinary C-C Motif Chemokine Ligand 14 (CCL14) for Persistent Severe Acute Kidney Injury. Kidney360.

[30] Ronco C., Tetta C., Mariano F., Wratten M.L., Bonello M., Bordoni V. (2003). Interpreting the mechanisms of continuous renal replacement therapy in sepsis: the peak concentration hypothesis. Artif Organs.

[31] Honoré P.M., Matson J.R. (2004). Extracorporeal removal for sepsis: acting at the tissue level–the beginning of a new era for this treatment modality in septic shock. Crit Care Med.

[32] Harm S., Falkenhagen D., Hartmann J. (2014). Pore size–a key property for selective toxin removal in blood purification. Int J Artif Organs.

[33] Cruz D.N., Antonelli M., Fumagalli R., Foltran F., Brienza N., Donati A. (2009). Early use of polymyxin B hemoperfusion in abdominal septic shock: the EUPHAS randomized controlled trial. Jama.

[34] Payen D.M., Guilhot J., Launey Y., Lukaszewicz A.C., Kaaki M., Veber B. (2015). Early use of polymyxin B hemoperfusion in patients with septic shock due to peritonitis: a multicenter randomized control trial. Intensive Care Med.

[35] Dellinger R.P., Bagshaw S.M., Antonelli M., Foster D.M., Klein D.J., Marshall J.C. (2018). Effect of targeted polymyxin B hemoperfusion on 28-day mortality in patients with septic shock and elevated endotoxin level: the EUPHRATES randomized clinical trial. Jama.

[36] Schädler D., Pausch C., Heise D., Meier-Hellmann A., Brederlau J., Weiler N. (2017). The effect of a novel extracorporeal cytokine hemoadsorption device on IL-6 elimination in septic patients: a randomized controlled trial. PLoS One.

[37] Lotz C., Heckelmann J., Lendzian C., Herrmann J., Haack B., Gieselmann M. (2026). Clinical and immunologic effects of extracorporeal cytokine removal in patients with septic shock: a randomized controlled trial. Shock.

[38] Klein D.J., Foster D., Walker P.M., Bagshaw S.M., Mekonnen H., Antonelli M. (2018). Polymyxin B hemoperfusion in endotoxemic septic shock patients without extreme endotoxemia: a post hoc analysis of the EUPHRATES trial. Intensive Care Med.

[39] Neyra J.A., Legrand M., Tidswell M.A., Al-Khafaji A., Galphin C., Rains R. (2026). Polymyxin B haemoadsorption in endotoxic septic shock (Tigris): a multicentre, open-label, Bayesian, randomised, controlled, phase 3 trial. Lancet Respir Med.

[40] Takeuchi T., Flannery A.H., Liu L.J., Ghazi L., Cama-Olivares A., Fushimi K. (2025). Epidemiology of sepsis-associated acute kidney injury in the ICU with contemporary consensus definitions. Crit Care.

[41] Stanski N.L., Rodrigues C.E., Strader M., Murray P.T., Endre Z.H., Bagshaw S.M. (2023). Precision management of acute kidney injury in the intensive care unit: current state of the art. Intensive Care Med.

[42] Sims C.R., Nguyen T.C., Mayeux P.R. (2016). Could biomarkers direct therapy for the septic patient?. J Pharmacol Exp Ther.

[43] Ostermann M., Ferrer R., Rimmelé T. (2025). Hemoadsorption in septic shock - PRO. Intensive Care Med.

[44] de Nooijer A.H., Pickkers P., Netea M.G., Kox M. (2023). Inflammatory biomarkers to predict the prognosis of acute bacterial and viral infections. J Crit Care.

[45] van der Poll T., van de Veerdonk F.L., Scicluna B.P., Netea M.G. (2017). The immunopathology of sepsis and potential therapeutic targets. Nat Rev Immunol.

[46] Stanski N.L., Wong H.R. (2020). Prognostic and predictive enrichment in sepsis. Nat Rev Nephrol.

[47] Bagshaw S.M., Al-Khafaji A., Artigas A., Davison D., Haase M., Lissauer M. (2021). External validation of urinary C-C motif chemokine ligand 14 (CCL14) for prediction of persistent acute kidney injury. Crit Care.

[48] Koyner J.L., Arndt C., Baldira Martinez de Irujo J., Coelho S., Garcia-Montesinos de la Peña M., di Girolamo L. (2024). Assessing the role of Chemokine (C-C motif) ligand 14 in AKI: a European consensus meeting. Ren Fail.

[49] Pickkers P., Darmon M., Hoste E., Joannidis M., Legrand M., Ostermann M. (2021). Acute kidney injury in the critically ill: an updated review on pathophysiology and management. Intensive Care Med.

[50] Zuzda K., Walczak-Wieteska P., Andruszkiewicz P., Małyszko J. (2025). Acute kidney injury biomarkers in perioperative care: a scoping review of clinical implementation. Diagnostics (Basel).

[51] Ostermann M., Lumlertgul N., Jeong R., See E., Joannidis M., James M. (2025). Acute kidney injury. Lancet.

