# Supplementary material for: The effects of hemoadsorption in patients with sepsis-associated persistent severe AKI who require CRRT: A prospective, randomized controlled pilot study
Source: Ann Intensive Care. 2026 Sep 22;16:100167. doi: 10.1016/j.aicoj.2026.100167 (PMC13656966; doi:10.1016/j.aicoj.2026.100167)
Supplement: Supplementary file 1 [file mmc1.docx]

**Table S1. Proportion and distribution of missing data for primary and secondary outcomes across study time points**

| **Outcomes** | **Time points** | **Total expected,**  **N** | **Total missing,**  **n (%)** | **Missing due to death within 72h, n(%)** | **Missing due to other reasons, n(%)** |
| --- | --- | --- | --- | --- | --- |
| Primary outcome |  |  |  |  |  |
| 30-day all-cause mortality | Day 30 | 76 | 0(0%) | 0(0%) | 0(0%) |
| Secondary outcomes |  |  |  |  |  |
| APACHE II Score | Baseline | 76 | 0(0%) | 0(0%) | 0(0%) |
|  | 72hours | 76 | 12(15.8%) | 12(15.8%) | 0(0%) |
| SOFA Score | Baseline | 76 | 0(0%) | 0(0%) | 0(0%) |
|  | 72hours | 76 | 12(15.8%) | 12(15.8%) | 0(0%) |
| Biomarkers | Baseline | 76 | 0(0%) | 0(0%) | 0(0%) |
|  | 72hours | 76 | 16(21.1%) | 12(15.8%) | 4(5.3%) |
| Cytokines | Baseline | 76 | 0(0%) | 0(0%) | 0(0%) |
|  | 72hours | 76 | 18(23.7%) | 12(15.8%) | 6(7.9%) |
| Hemodynamic Parameters | Baseline | 76 | 0(0%) | 0(0%) | 0(0%) |
|  | 72hours | 76 | 12(15.8%) | 12(15.8%) | 0(0%) |
| Duration of RRT | Discharge from ICU | 76 | 0(0%) | 0(0%) | 0(0%) |
| Duration of weaning from MV | Discharge from ICU | 76 | 0(0%) | 0(0%) | 0(0%) |
| Length of ICU stay | Discharge from ICU | 76 | 0(0%) | 0(0%) | 0(0%) |
| Length of hospital stay | Discharge from ICU | 76 | 0(0%) | 0(0%) | 0(0%) |
| Renal function prognosis |  |  |  |  |  |
| Recovery status within 7 days | Day 7 | 76 | 0(0%) | 0(0%) | 0(0%) |
| Requirement for RRT at discharge | Discharge from ICU | 76 | 0(0%) | 0(0%) | 0(0%) |
| Renal function recovery on Day 30 | Day 30 | 76 | 0(0%) | 0(0%) | 0(0%) |
| Persistent renal insufficiency on Day 30 | Day 30 | 76 | 0(0%) | 0(0%) | 0(0%) |

APACHE II, acute physiology and chronic health evaluation ii; SOFA, sequential organ failure assessment; RRT, renal replacement therapy; MV, mechanical ventilation; ICU, intensive care unit.

**Table S2. Levels of coagulation indices at enrollment and 72 h post-enrollment**

| **Coagulation indices** | **0h** | **72h** | **P value** |
| --- | --- | --- | --- |
| PT, s | 14.60(13.58-16.48) | 13.40(12.50-14.45) | 0.177 |
| TT, s | 15.35(14.58-16.98) | 16.25(14.97-18.10) | 0.811 |
| APTT, s | 32.85(29.90-35.88) | 31.80(27.93-39.00) | 0.581 |
| INR | 1.28(1.19-1.46) | 1.15(1.08-1.26) | 0.191 |
| FIB, g/l | 4.44(3.64-5.12) | 3.79(3.01-4.87) | 0.073 |

Data were presented as median (interquartile range, IQR). The P value indicate the statistical significance of differences between 0 h and 72 h, calculated using linear mixed-effects models (LMM). To satisfy model assumptions, all coagulation parameters were natural log-transformed prior to analysis.

PT, prothrombin time; TT, thrombin time; APTT, activated partial thromboplastin time; INR, international normalized ratio; FIB, fibrinogen.

| **Subgroup** | **CVVH+HA group** | | | **CVVH group** | | |  | |  | |  | |
| --- | --- | --- | --- | --- | --- | --- | --- | --- | --- | --- | --- | --- |
|  | **No of patients** | **30 day mortality**  **(95% CI)** |  | | **No of patients** | **30 day mortality**  **(95% CI)** | **Adjusted HR (95% CI)** | **Adjusted HR**  **(95% CI)** | | **P for interaction** | | **Adjusted HR (95% CI) for interaction** |
| **Age (years)** |  |  |  | |  |  |  |  | | 0.437 | | 1.73(0.43 to 6.92) |
| <=70 | 23 | 34.8%(16.4 to 57.3) |  | | 18 | 61.1%(35.7 to 82.7) |  | 0.40(0.16 to 1.02) | |  | |  |
| >70 | 15 | 40.0(16.3 to 67.7) |  | | 20 | 45.0%(23.1 to 68.5) |  | 0.72(0.25 to 2.06) | |  | |  |
| **Sex** |  |  |  | |  |  |  |  | | 0.710 | | 1.33(0.29 to 6.01) |
| Female | 11 | 36.4(10.9 to 69.2) |  | | 14 | 50.0(23.0 to 77.0) |  | 0.32(0.09 to 1.21) | |  | |  |
| Male | 27 | 37.0(19.4 to 57.6) |  | | 24 | 54.2(32.8 to 74.4) |  | 0.61(0.26 to 1.39) | |  | |  |
| **Diabetes mellitus** |  |  |  | |  |  |  |  | | 0.414 | | 2.00(0.38 to 10.64) |
| No | 15 | 34.8(16.4 to 57.3) |  | | 28 | 60.7(40.6 to 78.5) |  | 0.49(0.21 to 1.15) | |  | |  |
| Yes | 23 | 40.0(16.3 to 67.7) |  | | 10 | 30.0(6.7 to 65.2) |  | 1.48(0.32 to 6.82) | |  | |  |
| **Hypertension** |  |  |  | |  |  |  |  | | 0.371 | | 1.93(0.46 to 8.10) |
| No | 22 | 31.2(11.0 to 58.7) |  | | 21 | 61.9(38.4 to 81.9) |  | 0.35(0.12 to 1.01) | |  | |  |
| Yes | 16 | 40.9(20.7 to 63.6) |  | | 17 | 41.2(18.4 to 67.1) |  | 0.89(0.32 to 2.46) | |  | |  |
| **Apache II score** |  |  |  | |  |  |  |  | | 0.529 | | 1.66(0.34 to 7.99) |
| <=31 | 17 | 17.6(3.8 to 43.4) |  | | 22 | 40.9(20.7 to 63.6) |  | 0.33(0.09 to 1.25) | |  | |  |
| >31 | 21 | 52.4(29.8 to 74.3) |  | | 16 | 68.8(41.3 to 89.0) |  | 0.58(0.25 to 1.37) | |  | |  |
| **IL-6(pg/ml)** |  |  |  | |  |  |  |  | | 0.460 | | 0.58(0.13 to 2.49) |
| <1016.4 | 17 | 47.1(23.0 to 72.2) |  | | 21 | 57.1(34.0 to 78.2) |  | 0.67(0.27 to 1.68) | |  | |  |
| >1016.4 | 21 | 28.6(11.3 to 52.2) |  | | 17 | 47.1(23.0 to 72.2) |  | 0.23(0.07 to 0.81) | |  | |  |
| **CCL14(ng/ml)** |  |  |  | |  |  |  |  | | 0.009 | | 0.14(0.03 to 0.61) |
| <1.3 | 14 | 50.0(23.0 to 77.0) |  | | 15 | 33.3(11.8 to 61.6) |  | 1.92(0.60 to 6.15) | |  | |  |
| >1.3 | 24 | 29.2(12.6 to 51.1) |  | | 23 | 65.2(42.7 to 83.6) |  | 0.23(0.09 to 0.57) | |  | |  |
| **NGAL(ng/ml)** |  |  |  | |  |  |  |  | | 0.121 | | 3.35(0.73 to 15.43) |
| <177.0 | 17 | 23.5(6.8 to 49.9) |  | | 21 | 47.6(25.7 to 70.2) |  | 0.27(0.08 to 0.99) | |  | |  |
| >177.0 | 21 | 47.6(25.7 to 70.2) |  | | 17 | 58.8(32.9 to 81.6) |  | 0.96(0.38 to 2.40) | |  | |  |
| **KIM-1(ng/ml)** |  |  |  | |  |  |  |  | | 0.221 | | 2.59(0.56 to 11.91) |
| <3.1 | 22 | 31.8(13.9 to 54.9) |  | | 16 | 50.0(24.7 to 75.3) |  | 0.27(0.08 to 0.90) | |  | |  |
| >3.1 | 16 | 43.8(19.8 to 70.1) |  | | 22 | 54.5(32.2 to 75.6) |  | 0.73(0.27 to 2.02) | |  | |  |
| **Total** | 38 | 36.8(21.8 to 54.0) |  | | 38 | 52.6(35.8 to 69.0) |  | 0.53(0.26 to 1.07) | |  | |  |

**Table S3. Adjusted subgroup analysis of the effect of CVVH+HA versus CVVH on 30-day mortality**

The forest plot shows adjusted hazard ratios (aHRs) and 95% confidence intervals (CIs). Blue squares and the red diamond indicate point estimates for subgroups and the overall cohort, respectively. The vertical dashed line indicates an aHR of 1.0. P values for interaction indicate the heterogeneity of the treatment effect across strata. All models were adjusted for age, sex, and APACHE II score. The stratification variable itself was excluded from the adjustment within its respective subgroup to prevent collinearity.

CVVH, continuous veno-venous hemofiltration; HA, hemoadsorption; CI, confidence interval; APACHE II, Acute Physiology And Chronic Health Evaluation II; IL, interleukin; CCL14, chemokine (c–c motif) ligand 14; NGAL, neutrophil gelatinase-associated lipocalin; KIM-1, kidney injury molecule-1.

**Table S4. Proportional hazards assumption testing for the overall and subgroup Cox regression models**

| **Model / Subgroup Variable** | **P value** |
| --- | --- |
| Overall Cohort | 0.850 |
| Interaction Models |  |
| Age (years) | 0.978 |
| Sex | 0.849 |
| Diabetes mellitus | 0.876 |
| Hypertension | 0.312 |
| APACHE II score | 0.993 |
| IL-6 (pg/ml) | 0.131 |
| CCL14 (ng/ml) | 0.239 |
| NGAL (ng/ml) | 0.354 |
| KIM-1 (ng/ml) | 0.969 |

The proportional hazards (PH) assumption was evaluated using the Schoenfeld residuals test. A global P value ≥ 0.05 indicates that the PH assumption is met, confirming that the hazard ratios remain constant over the 30-day study period. The overall cohort model tested the main effect of the treatment (CVVH+HA versus CVVH alone). The interaction models tested the treatment effect along with its interaction with each corresponding subgroup variable.

PH, proportional hazards; CVVH, continuous veno-venous hemofiltration; HA, hemoadsorption; APACHE II, Acute Physiology And Chronic Health Evaluation II; IL, interleukin; CCL14, chemokine (c–c motif) ligand 14; NGAL, neutrophil gelatinase-associated lipocalin; KIM-1, kidney injury molecule-1.

**Table S5. Treatment effects on clinical scores, cytokines, biomarkers and hemodynamic parameters at the 72-hour endpoint derived from linear mixed-effects models**

| Indicator | Group | 72h EMM (95% CI) | Abs Red. | Rel Red. | P _group(72h)_ | P_interaction_ |
| --- | --- | --- | --- | --- | --- | --- |
| SOFA score | CVVH group | 10.39 (9.11 to 11.85) | 1.96 | 15.80% | 0.023 | 0.002 |
|  | CVVH+HA group | 8.36 (7.30 to 9.55) | 4.9 | 37.00% |  |  |
| APACHE II score | CVVH group | 19.93 (17.42 to 22.78) | 7.81 | 28.20% | 0.025 | 0.001 |
|  | CVVH+HA group | 16.02 (13.98 to 18.34) | 15.38 | 49.00% |  |  |
| CCL14(ng/ml) | CVVH group | 1.19 (0.74 to 1.76) | 0.3 | 19.90% | 0.312 | 0.125 |
|  | CVVH+HA group | 0.85 (0.46 to 1.34) | 0.94 | 52.40% |  |  |
| NGAL(ng/ml) | CVVH group | 72.56 (50.32 to 104.42) | 71.73 | 49.70% | 0.023 | 0.053 |
|  | CVVH+HA group | 132.95 (91.66 to 192.64) | 30.9 | 18.90% |  |  |
| KIM-1(ng/ml) | CVVH group | 2.49 (1.57 to 3.76) | 1.46 | 36.90% | 0.354 | 0.244 |
|  | CVVH+HA group | 3.31 (2.13 to 4.92) | 0.06 | 1.70% |  |  |
| IFN-γ(pg/ml) | CVVH group | 9.53 (5.70 to 15.57) | 3.78 | 28.40% | 0.989 | 0.678 |
|  | CVVH+HA group | 9.58 (5.62 to 15.92) | 6.66 | 41.00% |  |  |
| IFN-α(pg/ml) | CVVH group | 2.13 (1.81 to 2.48) | -0.36 | -20.30% | 0.076 | 0.041 |
|  | CVVH+HA group | 1.72 (1.43 to 2.04) | 0.17 | 9.00% |  |  |
| TNF-α(pg/ml) | CVVH group | 2.62 (1.98 to 3.41) | -0.67 | -34.30% | 0.183 | 0.009 |
|  | CVVH+HA group | 2.00 (1.45 to 2.67) | 0.51 | 20.40% |  |  |
| IL-1β(pg/ml) | CVVH group | 12.67 (8.42 to 18.84) | -1.09 | -9.40% | 0.045 | 0.083 |
|  | CVVH+HA group | 6.90 (4.38 to 10.62) | 4.73 | 40.60% |  |  |
| IL-10(pg/ml) | CVVH group | 3.41 (1.61 to 6.43) | 10.78 | 76.00% | 0.539 | 0.038 |
|  | CVVH+HA group | 2.49 (1.03 to 4.99) | 29.19 | 92.10% |  |  |
| IL-8(pg/ml) | CVVH group | 39.01 (22.57 to 66.93) | 75.19 | 65.80% | 0.266 | 0.25 |
|  | CVVH+HA group | 25.04 (14.09 to 43.95) | 94.8 | 79.10% |  |  |
| IL-5(pg/ml) | CVVH group | 2.64 (1.99 to 3.43) | -0.46 | -20.90% | 0.232 | 0.413 |
|  | CVVH+HA group | 2.07 (1.51 to 2.76) | 0.01 | 0.30% |  |  |
| IL-2(pg/ml) | CVVH group | 1.52 (1.05 to 2.10) | 0.08 | 5.00% | 0.697 | 0.083 |
|  | CVVH+HA group | 1.38 (0.92 to 1.94) | 0.89 | 39.30% |  |  |
| IL-17(pg/ml) | CVVH group | 5.54 (3.83 to 7.86) | -0.44 | -8.60% | 0.060 | 0.003 |
|  | CVVH+HA group | 3.31 (2.15 to 4.89) | 3.81 | 53.50% |  |  |
| IL-4(pg/ml) | CVVH group | 1.12 (0.96 to 1.30) | -0.06 | -6.10% | 0.633 | 0.945 |
|  | CVVH+HA group | 1.18 (1.01 to 1.37) | -0.07 | -6.50% |  |  |
| IL-12(pg/ml) | CVVH group | 1.28 (1.00 to 1.60) | -0.05 | -4.20% | 0.540 | 0.164 |
|  | CVVH+HA group | 1.15 (0.87 to 1.46) | 0.27 | 19.20% |  |  |
| IL-6(pg/ml) | CVVH group | 51.96 (28.15 to 95.24) | 443.78 | 89.50% | 0.235 | 0.016 |
|  | CVVH+HA group | 30.55 (16.01 to 57.52) | 1016.74 | 97.10% |  |  |
| MAP(mmHg) | CVVH group | 85.23 (80.22 to 90.24) | -7.23 | -9.30% | 0.168 | 0.009 |
|  | CVVH+HA group | 90.20 (85.19 to 95.22) | -19.02 | -26.70% |  |  |
| Lac(mmol/l) | CVVH group | 2.12 (1.66 to 2.65) | 1.17 | 35.60% | 0.295 | 0.023 |
|  | CVVH+HA group | 1.77 (1.36 to 2.24) | 2.34 | 57.00% |  |  |
| VIS | CVVH group | 7.21 (3.69 to 13.37) | 20.04 | 73.50% | 0.545 | 0.044 |
|  | CVVH+HA group | 5.44 (2.68 to 10.28) | 48.79 | 90.00% |  |  |
| Equivalent NE dose(ug/min) | CVVH group | 4.79 (2.52 to 8.54) | 12.71 | 72.60% | 0.594 | 0.039 |
|  | CVVH+HA group | 3.79 (1.91 to 6.88) | 30.29 | 88.90% |  |  |

Data are presented as estimated marginal means (EMMs) with 95% confidence intervals (CIs). EMMs and P values were generated using a linear mixed-effects model (LMM) to account for missing data and intra-subject correlations. Absolute reduction (Abs Red.) was calculated as the difference between the EMMs at baseline (0h) and 72h. Relative reduction (Rel Red.) was calculated as the percentage change from baseline. Negative reduction values indicate an increase in the parameter over time. P_group(72h)_ represents the statistical difference between the two treatment groups at the specific 72-hour time point. P_interaction_ denotes the group-by-time interaction effect, indicating whether the overall trajectory of change from 0h to 72h differed significantly between the CVVH and CVVH+HA groups.

Abbreviations: LMM, linear mixed-effects model; EMM, estimated marginal mean; CI, confidence interval; CVVH, continuous venovenous hemofiltration; SOFA, Sequential Organ Failure Assessment; APACHE II, Acute Physiology and Chronic Health Evaluation II; CCL14, chemokine (c–c motif) ligand 14; NGAL, neutrophil gelatinase-associated lipocalin; KIM-1, kidney injury molecule-1; IFN, interferon; TNF, tumor necrosis factor; IL, interleukin; MAP, mean arterial pressure; Lac, lactate; VIS, vasoactive inotropic score; NE, norepinephrine.
